# Biocontrol of citrus fungal pathogens by lipopeptides produced by *Bacillus velezensis* TZ01

**DOI:** 10.3389/fmicb.2024.1471305

**Published:** 2024-09-04

**Authors:** Baoju An, Danchao Du, Zhendong Huang, Zhanxu Pu, Jia Lv, Li Zhu, Shunmin Liu, Liping Zhang, Guoqing Chen, Lianming Lu

**Affiliations:** ^1^The Citrus Research Institute of Zhejiang Province, Taizhou, China; ^2^Key Laboratory of Fruit and Vegetable Function and Health Research of Taizhou, Taizhou, China

**Keywords:** *Bacillus velezensis*, antifungal activity, lipopeptides, citrus, fungal pathogens

## Abstract

Citrus diseases caused by fungal pathogens drastically decreased the yield and quality of citrus fruits, leading to huge economic losses. Given the threats of chemical pesticides on the environment and human health, biocontrol agents have received considerable attention worldwide as ecofriendly and sustainable alternative to chemical fungicides. In the present study, we isolated a *Bacillus velezensis* strain TZ01 with potent antagonistic effect against three citrus pathogenic fungi: *Diaporthe citri*, *Colletotrichum gloeosporioides* and *Alternaria alternata.* The culture supernatant of this strain exhibited remarkable antifungal activity on potato dextrose agar plates and detached leaves of five citrus varieties. Treatment with TZ01 culture supernatant obviously affected the hyphal morphology and caused nucleic acid leakage. The crude lipopeptides (LPs) extracted from the culture supernatant were found as the major active ingredients, and could maintain the activity under a wide range of temperature and pH and ultraviolet radiation. Furthermore, the type of LPs, produced *in vitro*, were explored. Whole-genome sequencing of TZ01 revealed secondary metabolite gene clusters encoding synthetases for non-ribosomal peptides and polyketide production, and gene clusters responsible for the synthesis of three important LPs (surfactin, iturin, and fengycin) were identified in the genome. The liquid chromatography-mass spectrometry analysis confirmed the presence of various homologs of surfactin A, bacillomycin D, and fengycin A in the extracted LPs. Taken together, these results contribute to the possible biocontrol mechanisms of *B. velezensis* strain TZ01, as well as providing a promising new candidate strain as a biological control agent for controlling citrus fungal pathogens.

## 1 Introduction

Citrus is one of the most popular fruits and is widely grown in over 140 countries throughout the tropical and subtropical regions. In addition to the nutritional value of citrus, the production circle also serves as a source of employment in many areas, thereby contributing to the economic development of citrus-producing areas. China accounts for approximately one-third of the world’s total citrus yield and planting area, and the growth of citrus production industry currently plays an important role in the agricultural economic development of China (Chaisiri et al., 2020).

Among the many factors that affect citrus production, such as climate change, cultivation technologies and fertilizers application, fungal pathogen that causes plant diseases is one of the most crucial factors that reduced the yield and quality of citrus fruits, leading to economic losses of world’s citrus-producing areas. Several important pathogenic fungi, such as *Diaporthe citri* (Xiong et al., 2021), *Colletotrichum gloeosporioides* (Wang et al., 2023), and *Alternaria alternata* (Peever et al., 2002), causes various diseases on citrus plant tissues and post-harvest fruits, including melanose, gummosis, stem end rot disease, anthracnose, and *Alternaria* brown spot. In addition to the reduction in citrus production, the low fruit quality, poor appearance, and shortened shelf-life of citrus fruits due to fungal infections also lead to severe commercial losses, particularly in China, where people prefer to consume fresh citrus fruits.

*Bacillus* spp. are Gram-positive bacteria with a rod-shape structure and inhabit different ecological niches such as soil, air, water, plants, and animals (Dutilloy et al., 2024). Currently, some strains of *Bacillus* spp. have shown potential as an effective bioagent for controlling citrus fungal pathogens because of their strong antifungal activity, broad spectrum antimicrobial properties, and environmental safety; thus, it is expected that the microbial preparations will gradually replace chemical pesticides application in future agricultural production (Bazioli et al., 2019; Kupper et al., 2020; Klein et al., 2016). The antifungal mechanism of *Bacillus* strains includes competition for resources; stimulation of plant defenses; and production of antifungal compounds such as enzymes, volatile compounds, and secondary metabolites (Khan et al., 2022). *Bacillus* strains are an important source of a wide spectrum of secondary metabolites, particularly those produced by nonribosomal peptide synthetases (NRPSs), polyketide synthases (PKSs), and hybride modular PKS-NRPSs (Dutilloy et al., 2024). For example, *Bacillus velezensis* FZB42 strain was the first gram-positive biocontrol bacteria whose genome was sequenced, and approximately 10% (340 kb) of its genome was found to be primarily reserved for the nonribosomal synthesis of lipopeptides (LPs), polyketide type antimicrobial molecules, siderophores, and bacteriocins (Chen et al., 2009).

LPs are low molecular weight cyclic or linear amphiphilic compounds consisted of β-amino or β-hydroxy fatty acids and oligopeptides (Toral et al., 2018). Because of their amphipathic structures, they possess a spectrum of antibacterial, antiviral, antifungal, and anticancer activities, which enable their use in a wide range of applications in agriculture, food production, medicine, and feed manufacturing (Saiyam et al., 2024). LPs are usually a mixture of homologues and isoforms that differ in fatty acid chain length and amino acids composition of the peptide sequences, which are the key factors that affect the biological activity of LPs (Juhaniewicz-Dȩbińska et al., 2020). Cyclic LPs mainly comprises of three families: surfactin and iturin, which are heptapeptides, and fengycin, which is a decapeptide (Akintayo et al., 2023). Iturin and fengycin possess strong antifungal activity against pathogenic filamentous fungi, while surfactin is known for its surface active properties and potent antimicrobial activities (Saiyam et al., 2024).

In the present study, a *Bacillus velezensis* strain TZ01 was identified and its antagonistic activity against *D. citri*, *C. gloeosporioides*, and *A. alternata* were evaluated *in vitro*. To better understand the biocontrol mechanisms of this strain, antifungal effects of the culture supernatant and subsequently extracted LPs from the culture supernatant were detected. Finally, whole-genome sequencing (WGS) was performed to identify putative gene clusters for secondary metabolites biosynthesis and liquid chromatography-mass spectrometry (LC-MS) analysis was used to explore antifungal substances in the LPs. This study will facilitate the development of biological strategies to control citrus fungal diseases and provide an alternative to conventional chemical treatment.

## 2 Materials and methods

### 2.1 Strains and plant materials

The fungal pathogens *Diaporthe citri*, *Colletotrichum gloeosporioides* and *Alternaria alternata* isolated from citrus orchard in Taizhou City (Zhejiang Province, China) were preserved in our laboratory and cultured on potato dextrose agar (PDA) plates (per liter: dextrose 20 g, boiling supernatant of 200 g potato, and agar 17 g) and potato dextrose broth (PDB) medium at 28°C. A *Bacillus*-like strain name TZ01 was isolated from the rhizosphere soil of a citrus tree in Taizhou City and was cultured in Luria-Bertani (LB) medium. Based on a previous method (Liu et al., 2022), this strain was identified by sequencing the *gyr*A gene with the following primers: gyrAF: 5′-CAGTCAGGAAATGCGTACGTCCTT-3′ and gyrAR: 5′- CAAGGTAATGCTCCAGGCATTGCT-3′.

Citrus leaves that have grown within one year were obtained from ten-year-old citrus trees, including Kashi No. 28 (*Citrus reticulata* Blanco), Ben Di Zao (*C. succosa* Hort. ex Tanaka), Huraka (*C. tamurana* Hort. ex Tanaka), and Grapefruit (*C. paradise* Macf.), and a twenty-year-old citrus tree which was Ponkan (*C. reticulata* Blanco). These citrus trees were planted in Taizhou City, Zhejiang province, China.

### 2.2 Dual culture assay on PDA plates

A dual culture assay on PDA plates was used to estimate the antagonistic activity of the TZ01 strain against *D. citri*, *C. gloeosporioides*, and *A. alternata*. Briefly, a mycelial plug with a diameter of 5 mm of these fungal species was placed at the center of a PDA plate, and the TZ01 strain was inoculated on the plate at 35 mm away from the mycelial plug. After culturing for 7 days post inoculation, the antagonistic effects of the TZ01 strain were assessed by observing the fungal colony growth.

### 2.3 Antagonism assay on citrus leaves

An antagonism assay on citrus leaves was conducted according to the method reported by Tanvir et al. (Ahmad et al., 2023). Leaves of the abovementioned citrus varieties were washed three times with sterilized water and then surface sterilized with 75% alcohol. The leaves were then punctured with a sterilized needle to create a wound with a diameter of 3 mm. *D. citri*, *C. gloeosporioides*, and *A. alternata* were cultured on PDA plates and the mycelium was punched out. A punched mycelial plug with a diameter of 3 mm of these fungi was inoculated onto the wound. Subsequently, the wound was treated with 10 μL sterile filtrate of the TZ01 culture supernatant or 10 μL LB in a control group. The leaves were then placed in a sterile plastic culture dish and cultured for 5 days at 28°C. The extent of leaf lesion and colony growth was observed to evaluate of fungal pathogenicity on leaves.

### 2.4 Extraction of crude LPs

Crude LPs were extracted by an acid precipitation method (Zhang Y. et al., 2022). *B. velezensis* TZ01 was cultured in 500 mL of LB medium at 30°C for 2 days with shaking at 200 rpm. The culture supernatant was collected by centrifugation at 8,000 rpm for 10 min. The pH value of the culture supernatant was adjusted to 2 by HCl and then incubated at 4°C overnight. The precipitates were collected by centrifugation and dissolved in methanol. Subsequently, the insoluble substances were removed by a second centrifugation at 12,000 rpm for 10 min. A rotary evaporator was used to evaporate the methanol and harvest the crude LPs. The dry LPs were dissolved in methanol after weighing.

### 2.5 Determination of the inhibition rate

The TZ01 strain was cultured in 100 mL LB medium for 2 days. A centrifugation at 8,000 rpm for 10 min was conducted to collect culture supernatant, followed by filtration with a 0.2 μm syringe filter. The sterile filtrate was added into a PDA plate at the final concentration of 10%, and a mycelial plug was placed at the center of the plate. A PDA plate containing 10% LB medium was used as a negative control. After culturing for 7 days at 28°C, the diameter of the fungal colony was measured. The inhibition ratio was determined as the following formula:


Inhibitionrate(%)=(C-T)/C×100%

where C means the fungal colony diameter on the control plate, T means the fungal colony diameter on the treated plate.

The inhibition rate of the crude LPs was determined using a method similar to that for the culture supernatant. Crude LPs were added into the PDA medium at the final concentrations at 1, 10, 20, 40, 50, 60, 80, 100, and 200 μg/mL. PDA plates containing the same volumes of methanol were used as a negative control group. After 7 days, the inhibition rate was measured and used to calculate the 50% inhibitory concentration of LPs against the tested fungal pathogens.

### 2.6 Microscopy analysis of hyphal morphology

The fungal pathogens were cultured in PDB medium and the sterile culture supernatant of TZ01 after a 24 h cultivation was added into the PDB medium at a ratio of 1:32. Changes in hyphal morphology were observed using a light microscope after 24 h of incubation.

### 2.7 Detection of hyphal nucleic acid leakage

*D. citri* was cultured in PDB medium, and the sterile culture medium of TZ01 was added into the PDB medium at a ratio of 1:4 and 1:32. The PDB medium containing same volume of LB was used as a control group. Then, the absorbance at OD_260_ of the PDB medium was measured at 0, 3, 6, 12, and 24 h of cultivation.

### 2.8 Stability test of LPs

Crude LPs were frozen at −20°C overnight, or incubated in water baths at 20°C, 40°C, 60°C, 80°C, and 100°C for 1 h. Subsequently, LPs were added into PDA plates at the final concentration of 60 μg/mL, and the inhibition rate against the tested fungi was measured after 7 days.

The pH value of LPs was adjusted to 1,3, 5, 7, 9, and 11 by using 2 M HCl or NaOH, and the LPs were then incubated at 4°C overnight. Subsequently, the pH of the LPs was adjusted to the original pH value, followed by the addition of the LPs into PDA plates.

A quartz glass material ultraviolet lamp with the rated power of 30 W was purchased from Sugeng Lighting Electrical Appliances Co., Ltd (Suzhou, China). LPs were exposed to UV light for 1 and 2 hours at a distance of 40 cm away from the ultraviolet lamp. The LPs were collected after irradiation and added into PDA plates.

### 2.9 Genome sequencing and annotation

After cultivation, the bacterial cells of *B. velezensis* TZ01 of were collected by a centrifugation at 8,000 rpm for 10 min. Subsequently, the cells were sent to BGI Genomics Company (Shenzhen, China) for whole genome sequencing (WGS) and annotation. Genome sequencing was performed using the platforms of Pacbio Sequel II and the DNBSEQ. The draft genomic unitigs were assembled using the Canu, which is a corrected circular consensus sequence subreads set. Gene prediction was performed on the TZ01 genome assembly by using glimmer3 system with Hidden Markov models. tRNAs, rRNAs and sRNAs were recognized with tRNAscan-SE, RNAmmer, and the Rfam database. Tandem Repeat Finder was used to annotate the tandem repeats. Functional annotation was performed by referring to the following seven databases: Kyoto Encyclopedia of Genes and Genomes (KEGG), cluster of orthologous groups (COG), Gene Ontology (GO), non-redundant (NR), Swiss-Prot, TrEMBL, and EggNOG.

The genome sequence data were also submitted to the antiSMASH database for identifying gene clusters for secondary metabolites biosynthesis and deposited in the NCBI website with GenBank accession number CP157671.

### 2.10 Liquid chromatography-mass spectrometry (LC-MS) analysis

The type of crude LPs was identified by LC-MS analysis using a LCMS-8050 mass spectrometer from Shinadzu (China) Co., Ltd (Kyoto city, Japan) according to the modified reported method (Pandey et al., 2023). The MS spectra was collected in the scan range 100–2000 *m/z*. The flow rate was 0.200 mL/min, and the sample was diluted 10-fold with methanol. The positive ion mode of electrospray ionization-mass spectrometry (ESI-MS) was used to acquire the mass spectra.

### 2.11 Statistical analysis

All experiments were performed at least three times independently. All statistical data were calculated with GraphPad Prism (Version 8.02). Statistical analysis was conducted using one-way ANOVA followed by Tukey’s multiple comparisons.

## 3 Results

### 3.1 Identification of the isolated strain

**FIGURE 1:**
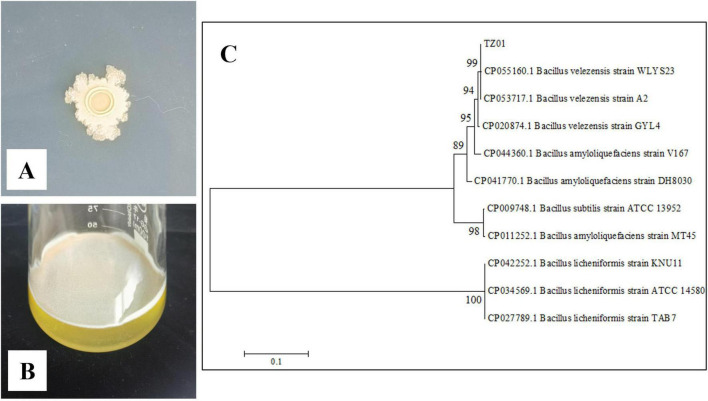
Morphological and molecular identification of isolated strain. **(A)** Colony morphology on the LB plate. **(B)** Biofilm formation after culturing in LB broth. **(C)** Construction of a phylogenetic tree of the isolated strain on the basis of the *gyr*A gene.

The isolated strain was cultured on a LB agar plate for 3 d. The colonies were light yellow in color with irregular margins and smooth surface (Figure 1A). These findings were consistent with the typical characteristics of the genus *Bacillus*. A thin film-like biofilm was observed on the surface of the liquid LB medium after culturing in a stationary phase for 1 d (Figure 1B), thus suggesting that this strain could produce surfactin (Zeriouh et al., 2014). A phylogenetic tree was built based on the *gyr*A gene sequence (Figure 1C), and the isolated strain and other *B. velezensis* strains were clustered into a branch; accordingly, we identified the strain as *B. velezensis* TZ01.

### 3.2 Strain TZ01 inhibits fungal pathogens growth

**FIGURE 2:**
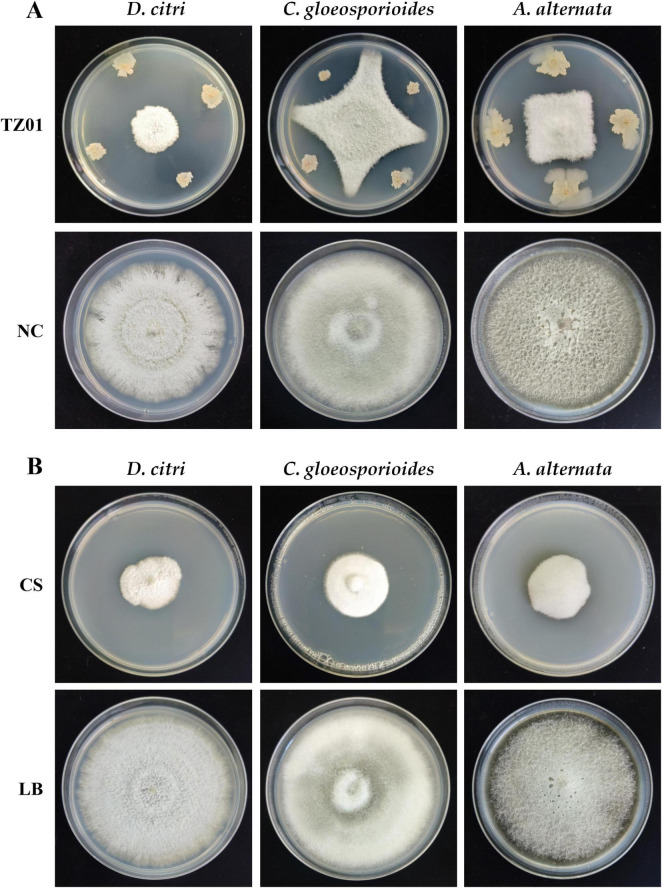
*B. velezensis* TZ01 inhibits fungal growth on PDA plates. **(A)** Confrontation culture of *B. velezensis* TZ01 with *D. citri*, *C. gloeosporioides*, and *A. alternata* for 7 days. In the control group, *D. citri*, *C. gloeosporioides*, and *A. alternata* were cultured on PDA plates alone. **(B)** *D. citri*, *C. gloeosporioides*, and *A. alternata* were cultured on PDA plates containing 10% TZ01 culture supernatant (CS) or LB medium for 7 days.

In the dual culture assay, the expansion of *D. citri*, *C. gloeosporioides*, and *A. alternata* colonies was stopped at a distance from the colony of *B. velezensis* TZ01; thus, TZ01 inhibited the growth of fungal colony (Figure 2A). Additionally, the distance between *B. velezensis* TZ01 and *D. citri* was more than those between the colonies of TZ01 and the other two fungal species; this might be due to the different sensitivities of these fungal species to the fungicidal compounds.

As the TZ01 strain inhibited fungal growth without direct contact with mycelia, we measured the inhibitory effects of the culture supernatant of the TZ01 strain on these fungal species. The fungal colony diameter was measured on PDA plates containing 10% culture supernatant or 10% LB medium after cultured for 7 days. The culture supernatant showed significant inhibitory effects against *D. citri*, *C. gloeosporioides*, and *A. alternata*, and the inhibition rates were 74.04% ± 2.52%, 65.62% ± 3.84% and 64.10% ± 4.18%, respectively (Figure 2B).

**FIGURE 3:**
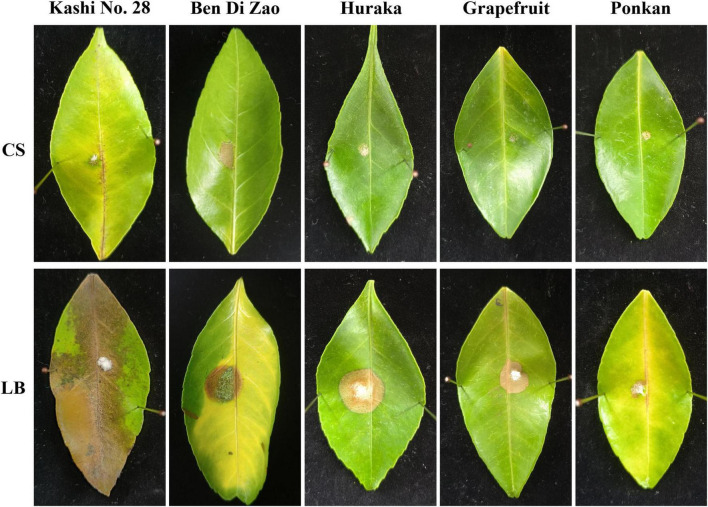
The culture supernatant of *B. velezensis* TZ01 inhibits *D. citri* growth on citrus leaves. *D. citri* was inoculated on the detached leaves of five citrus varieties and then treated with TZ01 culture supernatant (CS) in the experimental group or with LB medium in the control group. The names of the varieties are labeled above the images as follows: Kashi No. 28 (*Citrus reticulata* Blanco), Ben Di Zao (*C. succosa* Hort. ex Tanaka), Huraka (*C. tamurana* Hort. ex Tanaka), Grapefruit (*C. paradise* Macf.), and Ponkan (*C. reticulata* Blanco).

Antifungal effects of the TZ01 culture supernatant were also determined on detached citrus leaves of five varieties. After 5 days of cultivation on leaves, the colony expansion of *D. citri* treated with TZ01 culture supernatant was less than that of *D. citri* in the control group. Moreover, the experimental group showed alleviated leaf lesion than the control group, while the control group exhibited severe degree of leaf yellowing and apparent disease spots on leaf surface (Figure 3). The TZ01 culture supernatant also significantly inhibited the growth of *C. gloeosporioides* (Supplementary Figure 1) and *A. alternata* (Supplementary Figure 2) colonies on citrus leaves, with smaller disease spots on the leaves treated with the culture supernatant. The leaves treated with LB medium or TZ01 culture supernatant alone showed no disease spots on the wound area and no apparent changes in leaf appearance (Supplementary Figure 3).

### 3.3 TZ01 culture supernatant affects hyphal morphology and causes nucleic acid leakage of mycelium

**FIGURE 4:**
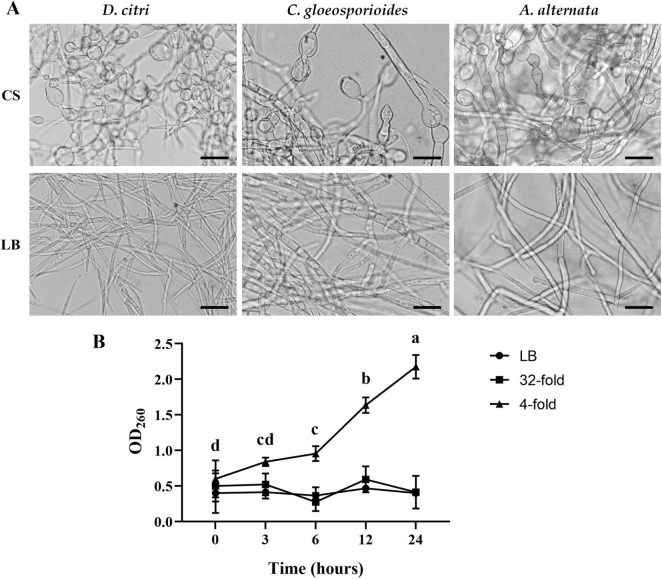
TZ01 culture supernatant induces changes in hyphal morphology and nucleic acid leakage. **(A)** *D. citri*, *C. gloeosporioides*, and *A. alternata* were treated with TZ01 culture supernatant (CS) or LB medium for 24 hours. Scale bar, 40 μm. **(B)** *D. citri* mycelium was cultured in PDB medium and TZ01 culture supernatant was added into the PDB medium at different concentrations (32-fold and 4-fold mean the ratio of TZ01 culture supernatant to PDB medium was 1:31 and 1:3, respectively).

*D. citri*, *C. gloeosporioides*, and *A. alternata* cultured in PDB medium were treated with the TZ01 culture supernatant for 24 h, and changes in hyphal morphology were observed by light microscopy. Morphological abnormalities such as hyphae swelling and formation of bulbs were commonly observed in the treated group (Figure 4A). In contrast, the hyphae treated with LB medium had a smooth surface, uniform morphology, and normal branches in the control group.

*D. citri* cultured in PDB medium was treated with TZ01 culture supernatant and changes in absorbance of the PDB medium was measured. At multiple time points, absorbance of PDB medium treated with TZ01 culture supernatant diluted 4-folds significantly increased, while the absorbance of PDB medium treated with TZ01 culture supernatant diluted 32-folds or LB medium remains almost unchanged (Figure 4B). Similar phenomenon of changes in absorbance was also observed in *C. gloeosporioides* and *A. alternata* (data not shown). These results suggested that higher concentration of TZ01 culture supernatant induced the leakage of *D. citri* mycelium. Lower concentration of TZ01 culture supernatant failed to cause any leakage of mycelium; however, hyphal morphology was affected. Therefore, we speculated that the antifungal mechanism of TZ01 culture supernatant changed dependent on its different concentrations.

### 3.4 LPs are the major antifungal components in the TZ01 culture supernatant

**FIGURE 5:**
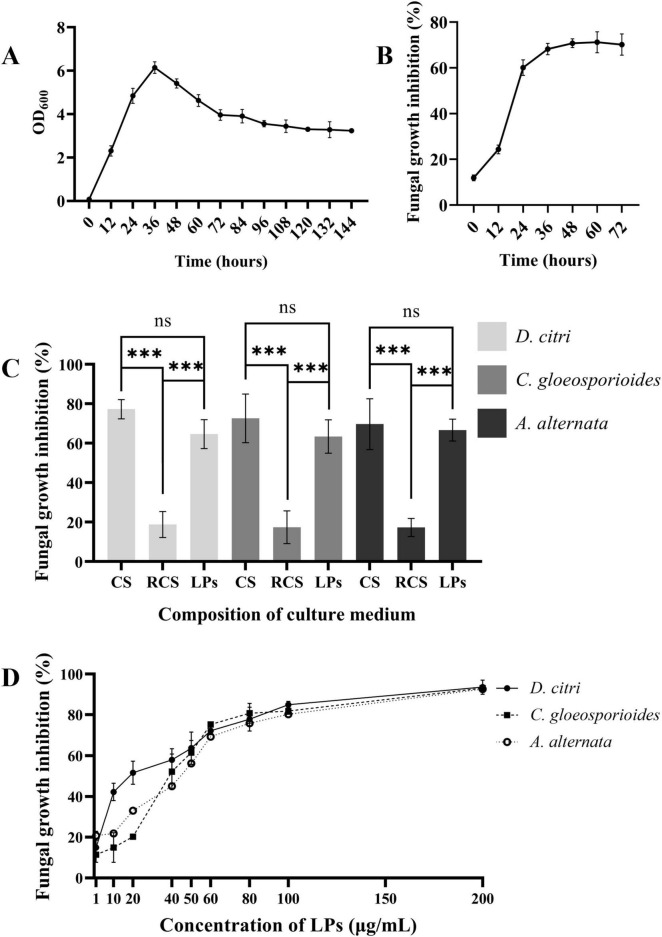
LPs are the major active components of the culture supernatant. **(A)** Growth curve of *B. velezensis* TZ01 in LB medium. **(B)** Changes in the inhibition rate of the culture supernatant against *D. citri* with cultivation time. **(C)** The inhibition rates of culture supernatant (CS), extracted LPs, and the residual culture supernatant (RCS) after extraction for *D. citri*, *C. gloeosporioides*, and *A. alternata*. **(D)** The inhibition rates of different concentrations of LPs against *D. citri*, *C. gloeosporioides*, and *A. alternata*. Data are expressed as the mean ± SD of three independent experiments; ns represents non-significant differences and *** represents *p* < 0.001.

The growth rate of the TZ01 strain in LB medium was detected by measuring the changes in the OD_600_ values in 144 h. The inhibitory effects of the TZ01 culture supernatant on *D. citri* were also simultaneously detected. According to the results, the TZ01 strain showed logarithmic growth in the first 36 h, and the bacterial density then slowly decreased (Figure 5A). The inhibitory effect of the culture supernatant on *D. citri* increased rapidly with the first 36 h of culture and then remained constant (Figure 5B). These results implied that the secretion of the antifungal components in the culture supernatant increased with bacterial proliferation.

LPs were extracted from the TZ01 culture supernatant followed by measuring their inhibitory effects against *D. citri*, *C. gloeosporioides*, and *A. alternata*. The inhibition rates of crude LPs were approximately 90% of that of the culture supernatant, indicating that the crude LPs were the major bioactive components secreted by the TZ01 strain (Figure 5C).

The inhibition rates of different concentrations of LPs were determined, and the 50% inhibitory concentration was calculated. The results showed that LPs at the concentration of 1 μg/mL had slight inhibitory effects, and as the concentration increased, the inhibition rates gradually elevated, suggesting that the antifungal activity of LPs was concentration-dependent (Figure 5D). When the concentration was less than 50 μg/mL, LPs showed higher inhibition rates against *D. citri* than *C. gloeosporioides* and *A. alternata*, thus implying that *D. citri* was more sensitive to the LPs. Based on the inhibition rates, the 50% inhibitory concentration of LPs against *D. citri*, *C. gloeosporioides* and *A. alternata* were 21.33 μg/mL, 51.40 μg/mL, and 56.49 μg/mL, respectively.

### 3.5 The LPs are thermally stable and resistant to acidic and alkaline pH and UV radiation

**FIGURE 6:**
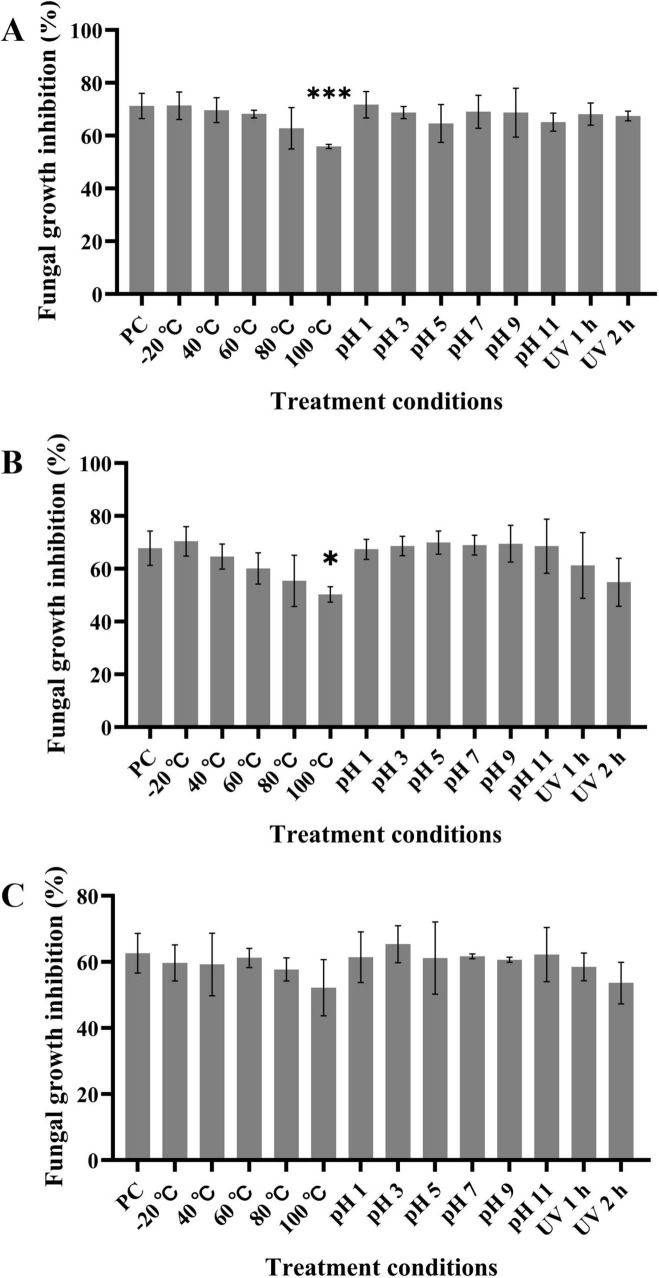
LPs shows thermal stability and resistance to acidic and alkaline pH and UV radiation. **(A–C)** Crude LPs at final concentration of 60 μg/mL were subjected to various treatments, and the inhibition rates of the treated LPs against *D. citri* **(A)**, *C. gloeosporioides* **(B)**, and *A. alternata* **(C)** were measured. PC represents the inhibition rate of untreated LPs (pH 7, 20°C, and non-UV radiation). Data are expressed as the mean ± SD of three independent experiments. Compared to the PC column, columns without asterisks above them represent non-significant differences; * represents *p* < 0.05, and *** represents *p* < 0.001.

The effects of temperature, pH, and UV radiation on the LPs antagonistic activity to *D. citri* (Figure 6A), *C. gloeosporioides* (Figure 6B), and *A. alternata* (Figure 6C) were assessed. LPs at 60 μg/mL showed a stable activity in a wide range of temperatures from −20°C to 80°C; however, the inhibitory activity decreased at 100°C. Furthermore, the inhibitory activity of LPs remained almost unaffected in the pH range of 1 to 11. There was no significantly change of LPs activity after 1 or 2 h of UV radiation exposure. Thus, LPs have favorable characteristics of thermal stability and resistance to acidic and alkaline pH and UV radiation, which support their further applications.

### 3.6 Gene clusters associated with secondary metabolites in the TZ01 genome

**FIGURE 7:**
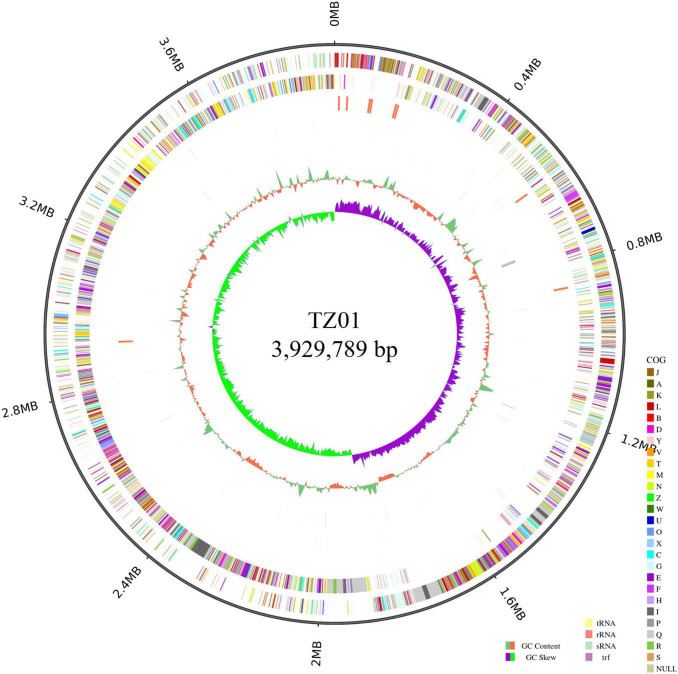
Circular genome of *B. velezensis* TZ01. From the outer ring to the inner ring: ring 1 represents genome size, ring 2 represents forward strand genes colored according to the cluster of orthologous groups (COG) classification, ring 3 represents reverse strand genes colored according to the cluster of orthologous groups (COG) classification, ring 4 represents forward strand ncRNA, ring 5 represents reverse strand ncRNA, ring 6 represents repeat positions, ring 7 represents GC content, and ring 8 represents GC skew value.

We analyzed the genome of the TZ01 strain to investigate the genetic basis of its antagonistic property (Figure 7). The TZ01 genome consists of a circular chromosome of 3,929,789 bp in size, and the average GC content is 46.49%. The chromosome was predicted to contain 3,959 genes, 86 tRNAs, 27 rRNAs, and 31 sRNAs. According to the GO annotation results, 3,836, 85, and 2,776 genes were found to be involved in three main parts of biological process, cellular component and molecular function, respectively (Supplementary Figure 4). The COG analysis revealed that 3,445 genes were classified into 24 different functional categories (Supplementary Figure 5). The top 5 functional categories with clear functions were amino acid transport and metabolism (305 genes); transcription (286 genes); carbohydrate transport and metabolism (259 genes); cell cycle control, cell division and chromosome partitioning (213 genes); and translation, ribosomal structure and biogenesis (211 genes). A total of 97 (2.82%) genes were listed in the functional category of secondary metabolites biosynthesis, transport, and catabolism. Additionally, KEGG annotation predicted that 2,800 genes were related to 42 different subparts, which constituted 6 main parts of the biological pathways (Supplementary Figure 6).

The genome sequence data were subsequently submitted to the antiSMASH database, and 12 gene clusters associated with secondary metabolites synthesis were identified in the genome (Table 1). The NRPSs associated with surfactin synthesis were encoded by synthetic genes in cluster 1. Clusters 5 and cluster 10 encoded PKSs for macrolactin H and difficidin synthesis, respectively. Cluster 6 encoded a hybrid modular PKS-NRPSs for bacillaene synthesis. Although cluster 7 was predicted to generate NRPSs responsible for fengycin synthesis, in-depth mining revealed that two main NRPSs operons were responsible for the biosynthesis of fengycin and bacillomycin D, which is another member of the iturin group (Sui et al., 2022). Cluster 11 was predicted to be involved in bacillibactin synthesis. Thus, the diverse gene clusters associated with secondary metabolites synthesis revealed the genetic basis for the high antifungal activity of the LPs from TZ01 strain.

**TABLE 1:** Analysis of *B. velezensis* TZ01 gene clusters associated with secondary metabolites.

Cluster	Type	Size (bp)	Most similar known cluster	Similarity
1	NPRS^1^	65,408	Surfactin	82%
2	PKS-like^2^	41,245	Butirosin A/butirosin B	7%
3	Terpene	20,741	–	–
4	Lanthipeptide-class-ii	28,889	–	–
5	transAT-PKS	88,234	Macrolactin H	100%
6	transAT-PKS^3^, T3PKS^4^, NRPS	110,094	Bacillaene	100%
7	NRPS, transAT-PKS, betalactone	137,802	Fengycin	100%
8	Terpene	21,884	–	–
9	T3PKS	41,101	–	–
10	transAT-PKS	106,183	Difficidin	100%
11	NRP-metallophore, NRPS, RiPP-like^5^	51,792	Bacillibactin	100%
12	Other	41,419	Bacilysin	100%

^1^NRPS, nonribosomal peptide synthetase;

^2^PKS, polyketide synthase;

^3^transAT-PKS, trans-acyltransferase polyketide synthase;

^4^T3PKS, type III polyketide synthase;

^5^RiPP-like, post-translationally modified peptide-like ribosomally synthesized and post translationally modified peptide-like.

### 3.7 Crude LPs comprise surfactin A, bacillomycin D, and fengycin A

**FIGURE 8:**
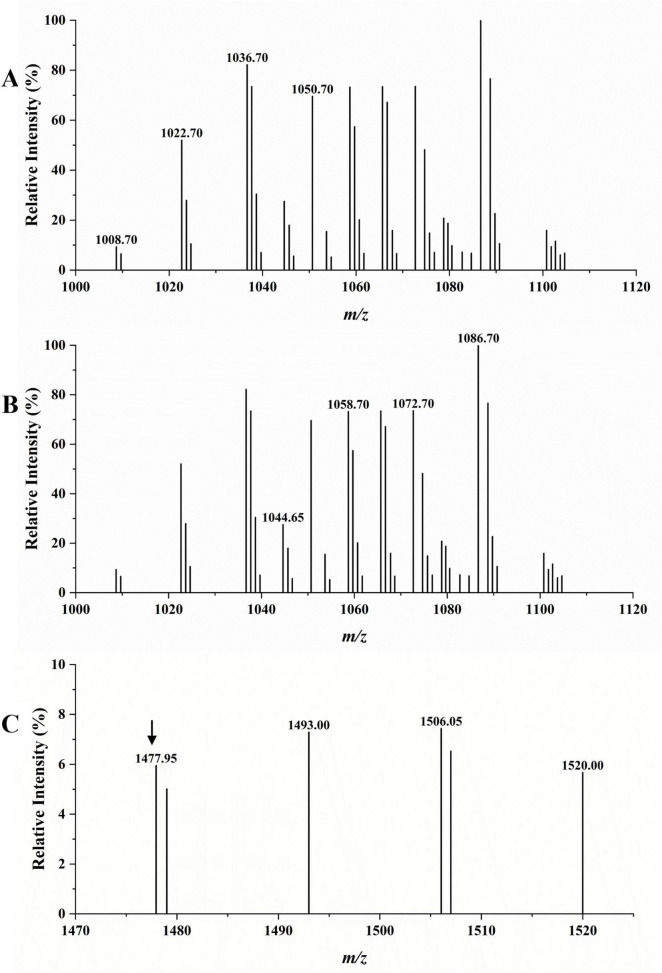
LC-MS analysis of crude LPs. **(A)** Surfactin A (marked with specific *m/z*). **(B)** Bacillomycin D (marked with specific *m/z*). **(C)** Fengycin A (marked with an arrow above it).

The LC-MS analysis was used to detect the type of the extracted crude LPs. According to the results in Figure 8, in the scanning spectrum of *m/z* 100–2,000, the molecular ion peaks [M + H^+^] at *m/z* 1008.70, 1022.70, 1036.70, and 1050.70 exhibited a 14 Da (-CH_2_) difference in molecular weights, and might correspond to the reported C_13–16_ surfactin A, respectively (Cao et al., 2018). Similarly, molecular ion peaks [M + H^+^] at *m/z* 1044.65, 1058.70, 1072.70, and 1086.70 also exhibiting a 14 Da difference in molecular weights might correspond to the reported C_14–17_ bacillomycin D (Jiao et al., 2021). The peak at *m/z* 1477.95 was attributed to the protonated molecular ions of C_17_ fengycin A (Zhang D. et al., 2022). Therefore, the MS results confirmed the presence of surfactin, bacillomycin D, and fengycin in the extracted LPs, consistent with the genome sequencing results.

## 4 Discussion

For the purpose of protecting citrus plants and fruits quality, many *Bacillus* species have been tested for their antagonistic effects on the basis of produced secondary metabolites or as plant endophytic bacteria against fungal pathogens *Penicillium* spp. (Wang et al., 2024), *C. gloeosporioides* (Vu et al., 2023), *C. acutatum* (Klein et al., 2016), and *Phyllosticta citricarpa* (Kupper et al., 2020), and other non-fungal pathogens, such as *Xanthomonas citri* subsp. *citri* that causes citrus canker disease (Rabbee and Baek, 2023) and *Candidatus Liberibacter asiaticus* that causes devastating Huanglongbing disease (Munir et al., 2022). Presently, the protected cultivation mode has become a popular planting mode in many southern regions of China. This approach utilizes a plastic greenhouse to deal with the issues of winter freezing injuries and pest invasions and simultaneously promote fruits maturity and quality improvement. However, because the greenhouse environment is also suitable for the growth and spread of fungal pathogens, this cultivation approach led to an increasing occurrence of some fungal diseases. Therefore, the isolation of *Bacillus* species with an excellent antifungal activity is crucial for the biological control of the increasing incidence of fungal diseases in citrus.

In the present study, we tested the antagonistic effects of the TZ01 culture supernatant on citrus leaves. However, the symptoms of the leaves inoculated with these fungi were different from those of plant diseases induced by these fungal pathogens in the natural environment. This might be due to the absence of long-term latency of pathogens and interactions between parasitic effects or secreted toxins of fungi with the plant immune systems in our experiments (Akimitsu et al., 2003). Therefore, we mainly measured fungal growth on leaves as well as the degree of leaf lesions, according to a previously reported method (Dutilloy et al., 2024). Among the three tested fungal pathogens, *D. citri* caused more apparent disease spots on leaves than *C. gloeosporioides* and *A. alternat*; this might be due to differences in the rate of infection or different sensitivities of citrus plants to the fungal pathogens (Chaisiri et al., 2022). Moreover, the citrus variety Kashi No. 28 appeared to be more susceptible to these fungi because of the larger disease spots and more apparent changes in leaf appearance.

By performing WGS, we predicted the gene clusters of various secondary metabolites in TZ01 genome. The LC-MS analysis detected the presence of homologs of LPs in TZ01 culture supernatant (Hu et al., 2024). Different *Bacillus thuringiensis* strains can produce multiple combinations of LPs types or various homologs or isoforms of one kind LP, and therefore, the antifungal effects of these strains will also change accordingly. Two *B. velezensis* strains Y6 and Y7 producing three homologs of iturin, five homologs of fengycin, and five homologs of surfactin showed antagonistic effect against *Ralstonia solanacearum* and *Fusarium oxysporum* (Cao et al., 2018). *B. amyloliquefaciens* strain YN201732 with biocontrol activity against *Erysiphe cichoracearum* could produce three homologs of bacillomycin D, and each homologs contained several isoforms (Jiao et al., 2021). *B. subtilis* strain ZD01, with antifungal activity against *Alternaria solani* in potato, could produce three homologs of surfactin and five homologs of fengycin (Zhang D. et al., 2022). In the present study, various homologs of surfactin A and bacillomycin D were detected as the major components in LPs produced by strain TZ01, and the extracted LPs could effectively inhibit the growth of citrus fungal pathogens. Therefore, this research provides a basis for understanding the antagonistic effects of the combined usage of surfactin A and bacillomycin D on citrus fungal pathogens, especially on *D. citri*, which could almost invade all citrus varieties (Chaisiri et al., 2022). At the meantime, the threatens of *D. citri* on citrus production increase, but there is few research on its biological managements. Here, we reported that the inhibitory rate of strain TZ01 exceeds 70% against *D. citri*, suggesting that TZ01 has the potential for biocontrol of citrus fungal diseases. Apart from fengycin A, peaks at *m/z* 1493.00 and 1506.05 were very close to the homologs of fengycin B; therefore, these peaks could be attributed to C_16_ and C_17_ fengycin B (Ali et al., 2015). However, these findings require further confirmation. Moreover, there was other molecular ion peaks detected, implying the possibility of presence of bacillaene, macrolactin H, difficidin, or bacillibactin, which requires further exploration (Figure 8 and Supplementary Figure 7).

The light microscope observation revealed a common morphological change in the hyphae of the fungal pathogens, with some swollen structures appearing at the tip and in the central part of the treated hyphae. Similar phenomena were also induced by bacillomycin D and fengycin from *B. amyloliquefaciens* LYZ69 (Hu et al., 2021) and iturin from *B. amyloliquefaciens* 41B-1 (Gu et al., 2017). Treatment with LPs activated the HOG signaling pathway, leading to the rapid accumulation of glycerol, followed by changes in the osmotic pressure in hyphal cells and the appearance of swollen structures. In addition, Hoechst 33258 and PI staining demonstrated the occurrence of chromatin condensation and destruction of plasma membrane integrity in the swollen structures, which indicated that LPs induced cell apoptosis (Hu et al., 2021). Therefore, the swollen structure suggested that the LPs secreted by strain TZ01 could induce signaling pathway activation and cell apoptosis in hyphae. An interesting phenomenon was also observed that the swollen structure appeared when the TZ01 culture supernatant was diluted between 8 and 32 times, while no swollen structure appeared when the culture supernatant was diluted 4 times or less (data not shown). Therefore, cell signaling pathway activation and cell apoptosis only occurs at appropriated concentrations of LPs.

It is known that LPs such as surfactin, iturin, and fengycin can insert into the cell membrane through their hydrophobic tails after binding to the membrane, leading to increased local membrane curvature and instability, followed by transient membrane disruption or local bilayer disorder, which results in changes in cell permeability (Balleza et al., 2019). The treatment of crude LPs to *A. alternata* mycelium resulted in the efflux for intracellular ions and elevated extracellular conductivity, suggesting the increased permeability of fungal cell membrane (Zhang et al., 2024). In our experiment, the hyphal leakage experiment was performed using extracted LPs; however, the solubility of crude LPs decreased, and we were unable to detect mycelial leakage by LPs. Therefore, TZ01 culture supernatant was used and changes in hyphal morphology and nucleic acid leakage were determined. In the present study, higher concentration of TZ01 culture supernatant caused hyphal nucleic acid leakage; however, lower concentration of culture supernatant induced hyphal swollen structure appeared, with no hyphal leakage. Based on these results, we speculated that there might be different mode of action depending on different LPs concentrations, and lower concentration of LPs might exert its adverse effects on mycelium by activation of certain signaling pathways, through hardly caused membrane leakage. The next step of our research will explore the contribution of membrane insertion and induction of cell signaling pathways to LPs antifungal effects.

## 5 Conclusion

The present study showed that *Bacillus velezensis* strain TZ01 demonstrated potent antifungal activity against *D. citri*, *C. gloeosporioides*, and *A. alternata*, which leading to citrus fungal diseases. The secreted antifungal substances affected hyphal morphology and cause cell membrane damage, leading to the inhibition of fungal growth. The LPs in the culture supernatant were proved to be the major active ingredients for the antagonistic action, and surfactin A, bacillomycin D, and fengycin A were detected through WGS and LC-MS analysis. In summary, this study explored the mechanism of action of the *Bacillus velezensis* strain TZ01 and suggested that TZ01 had great potential as a promising biocontrol agent for controlling the citrus fungal diseases.

## Data Availability

The datasets presented in this study can be found in online repositories. The names of the repository/repositories and accession number(s) can be found in the article/Supplementary material.
