# Supplementary material for: Biocontrol of citrus fungal pathogens by lipopeptides produced by Bacillus velezensis TZ01
Source: Front Microbiol. 2024 Sep 4;15:1471305. doi: 10.3389/fmicb.2024.1471305 (PMC11408202; doi:10.3389/fmicb.2024.1471305)
Supplement: Supplementary file 1 [file Data_Sheet_1.DOCX]

Supplementary Material

Figure S1. Culture supernatant of *B. velezensis* TZ01 inhibits *C. gloeosporioides* growth on citrus leaves.

Figure S2. Culture supernatant of *B. velezensis* TZ01 inhibits *A. alternata* growth on citrus leaves.

Figure S3. Citrus leaves are not affected by the TZ01 culture supernatant (CS) and LB medium.

Figure S4. GO analysis of the genome of *B. velezensis* TZ01 genome.

Figure S5. COG analysis of the genome of *B. velezensis* TZ01 genome.

Figure S6. KEGG analysis of the genome of *B. velezensis* TZ01 genome.

Figure S7. Molecular ion peaks of LPs [M + H^+^] at *m/z* 200-700.
