# Supplementary figures and images for: Biocontrol of citrus fungal pathogens by lipopeptides produced by Bacillus velezensis TZ01
Source: Front Microbiol. 2024 Sep 4;15:1471305. doi: 10.3389/fmicb.2024.1471305 (PMC11408202; doi:10.3389/fmicb.2024.1471305)

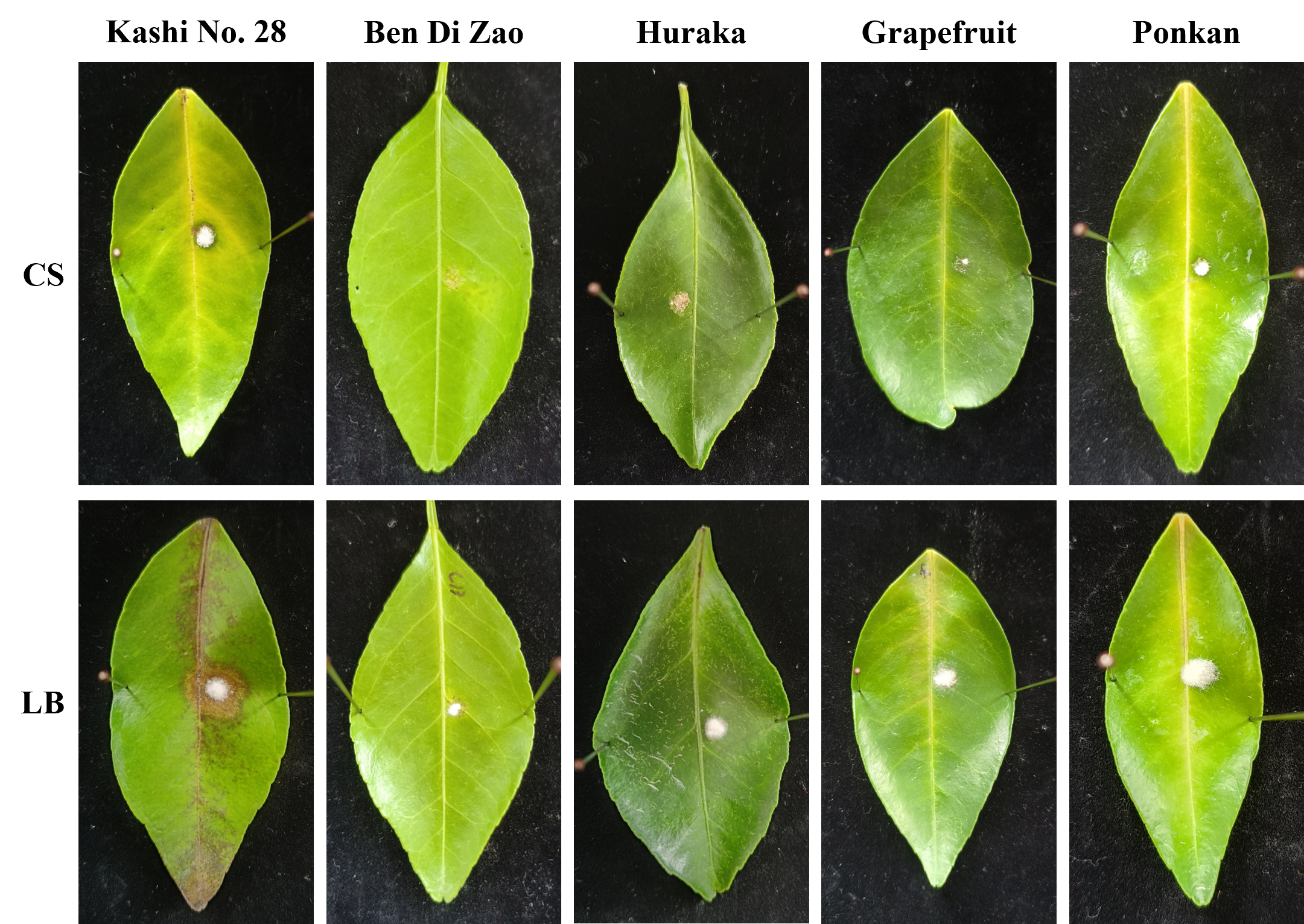

Supplement: Supplementary file 2 [file Image_1.TIF]

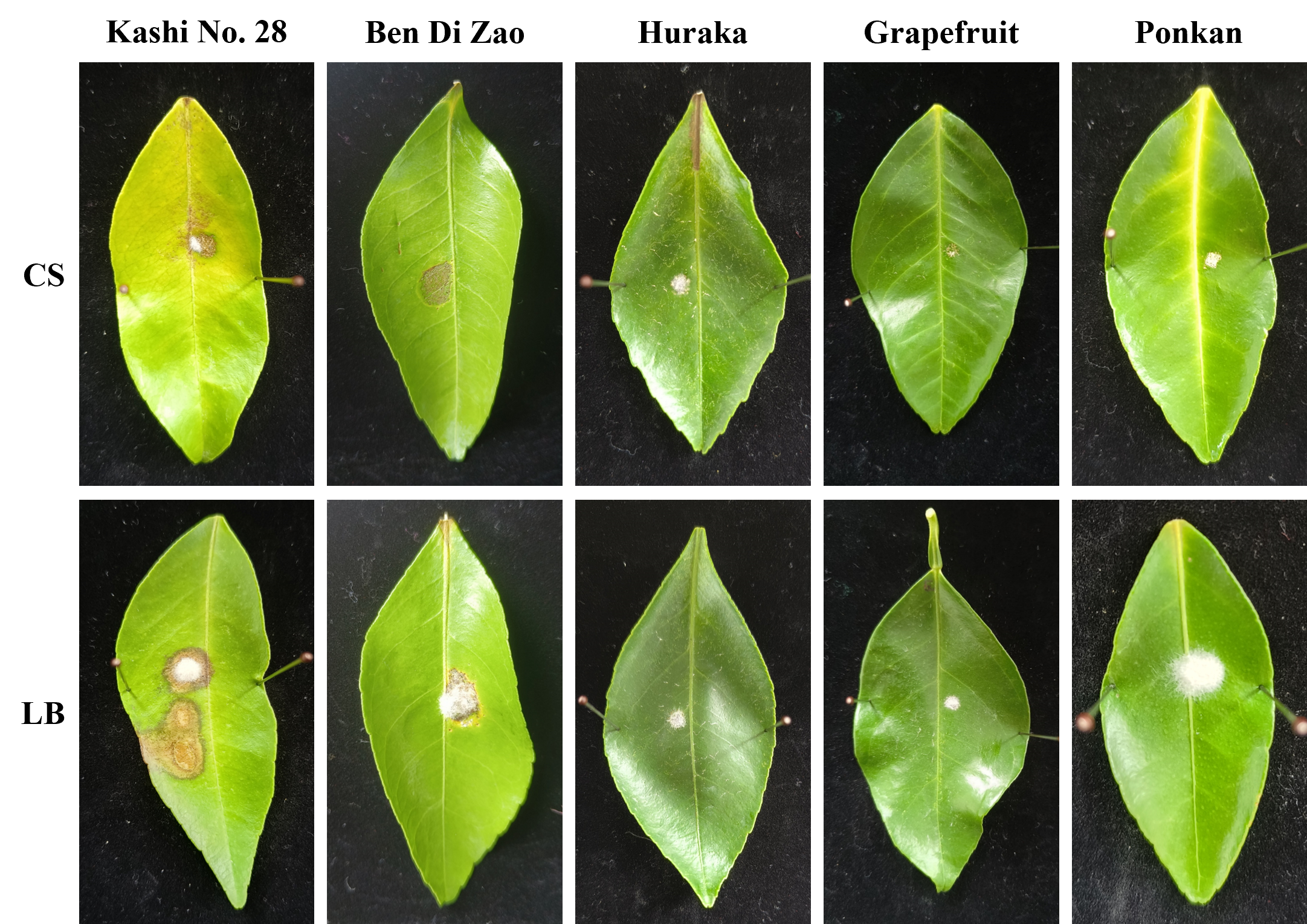

Supplement: Supplementary file 3 [file Image_2.TIF]

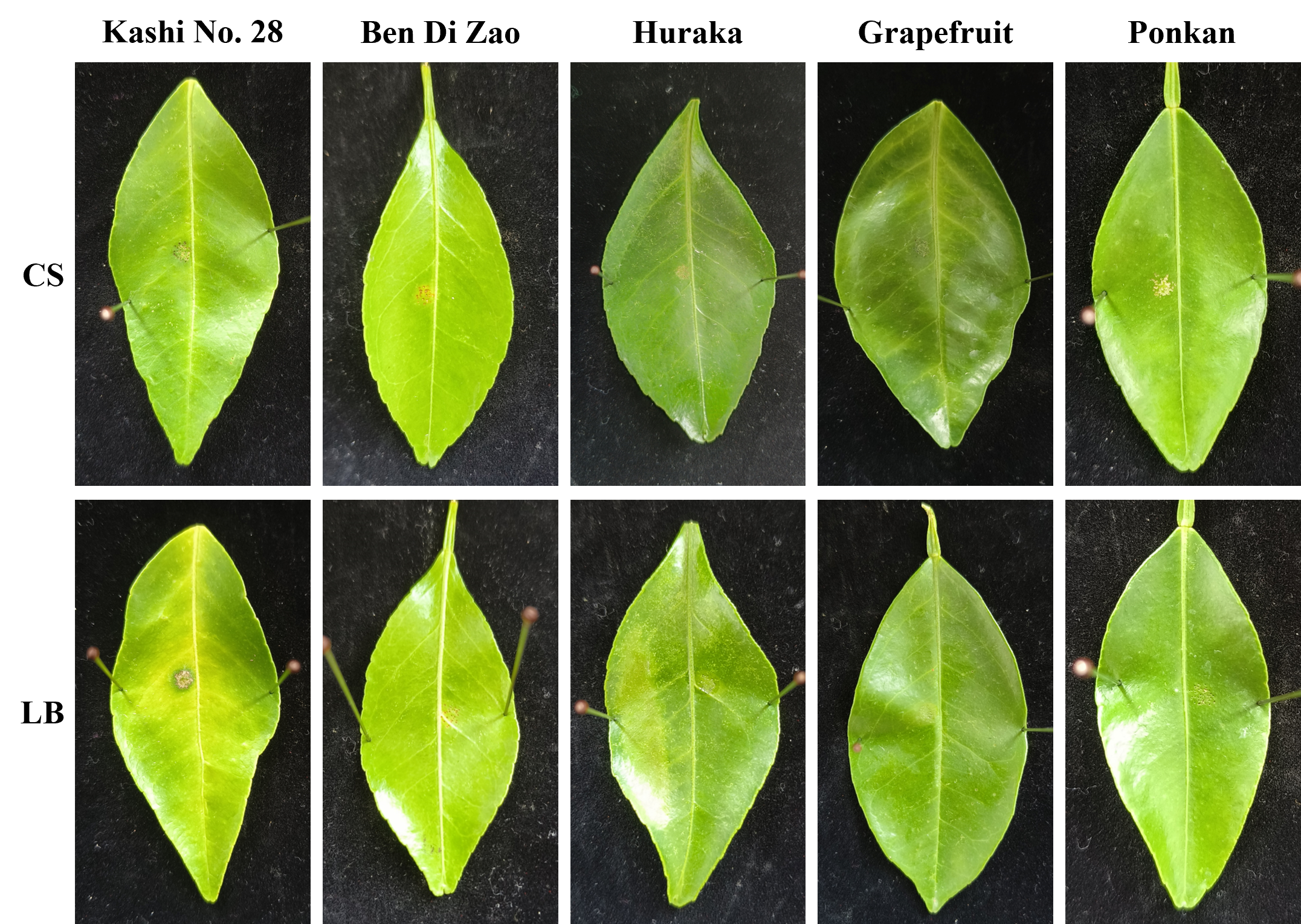

Supplement: Supplementary file 4 [file Image_3.TIF]

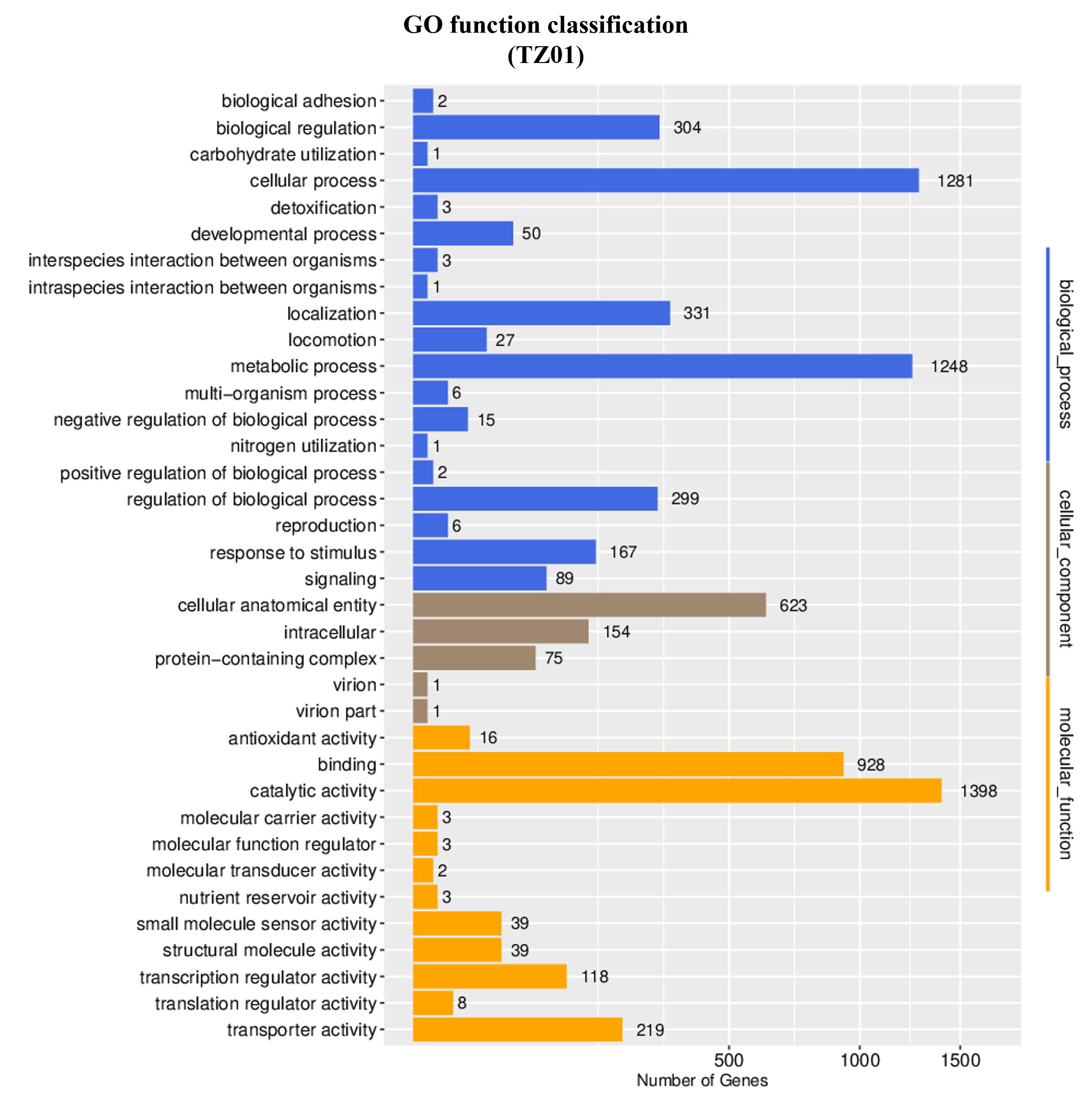

Supplement: Supplementary file 5 [file Image_4.TIF]

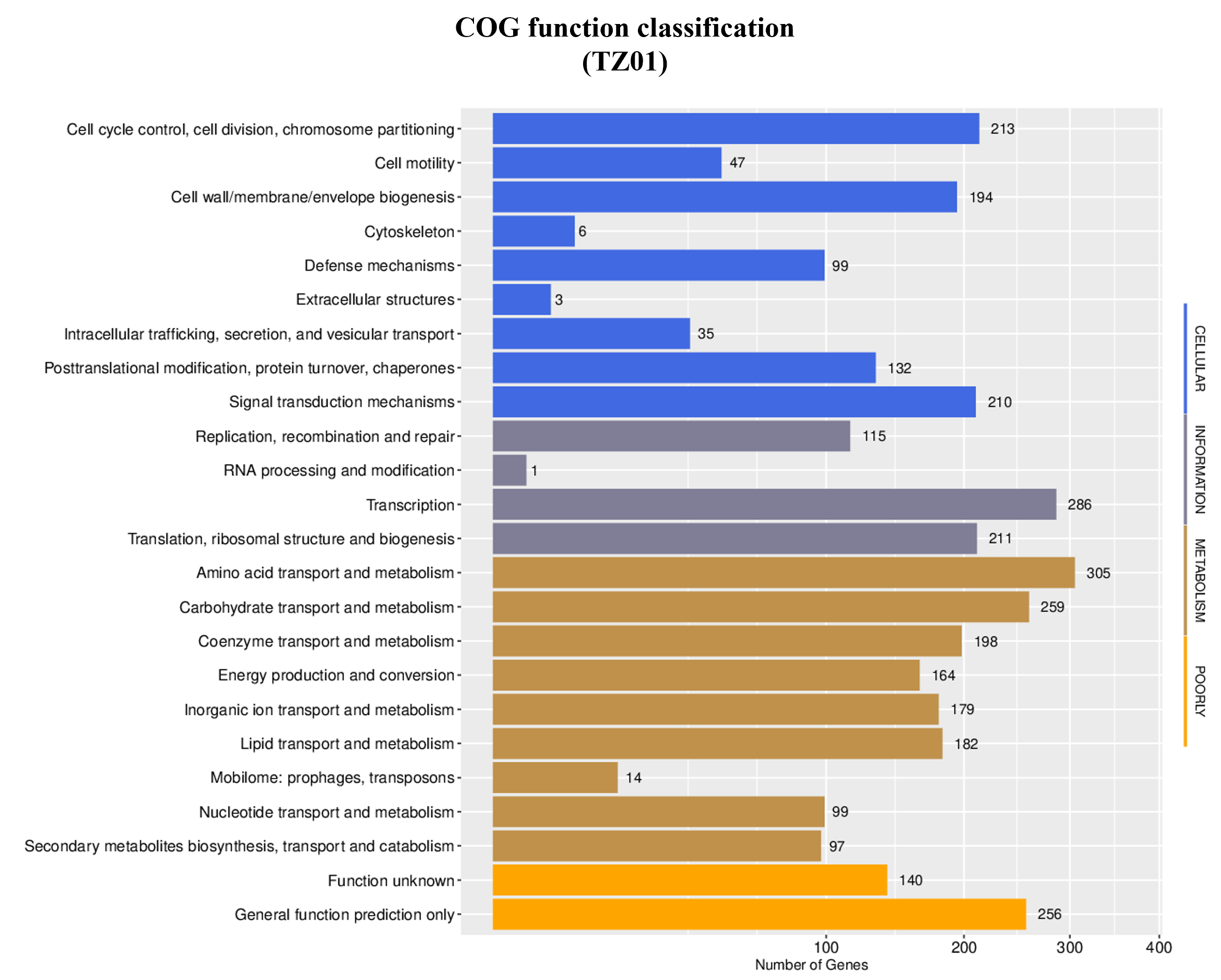

Supplement: Supplementary file 6 [file Image_5.TIF]

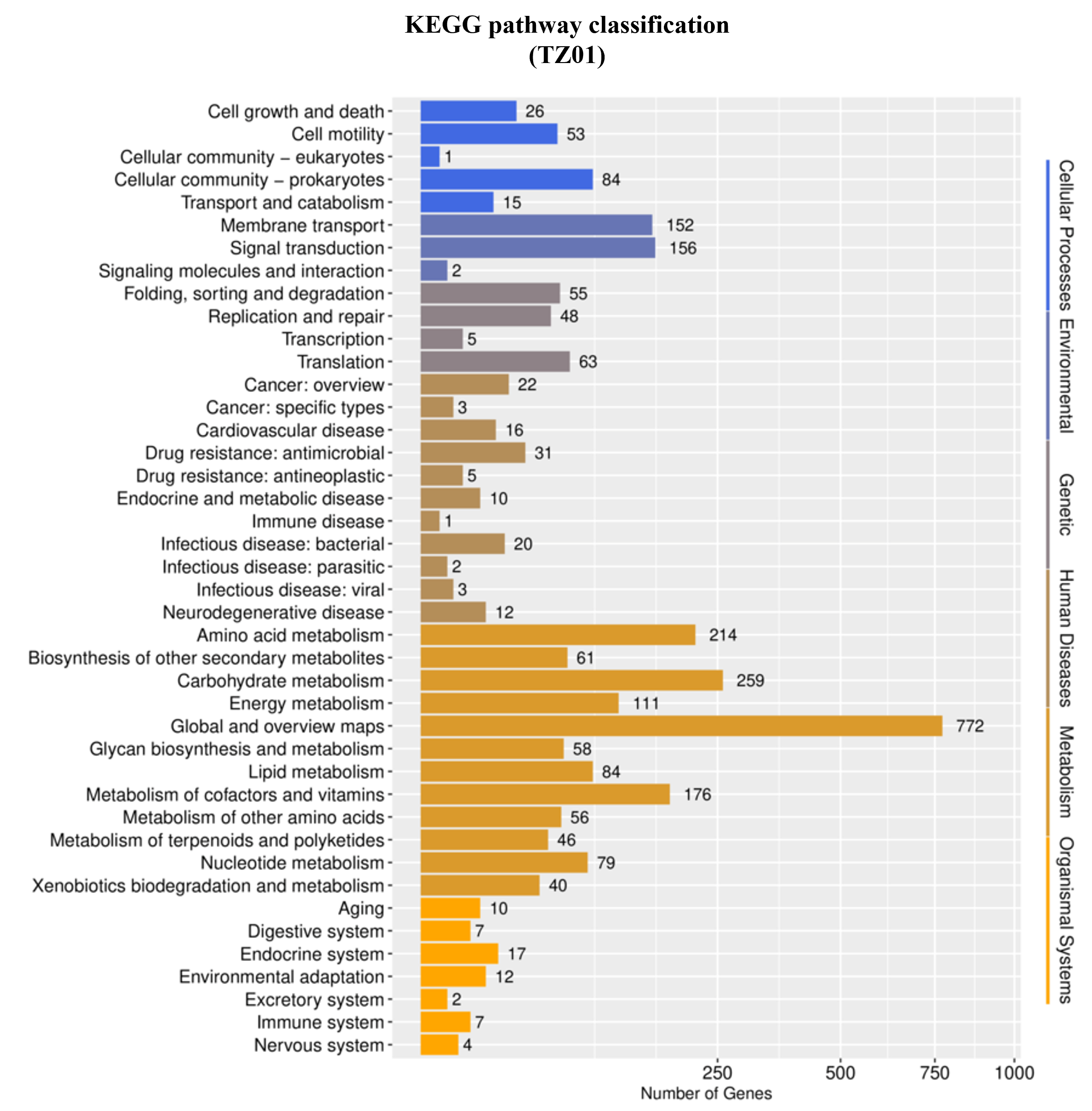

Supplement: Supplementary file 7 [file Image_6.TIF]

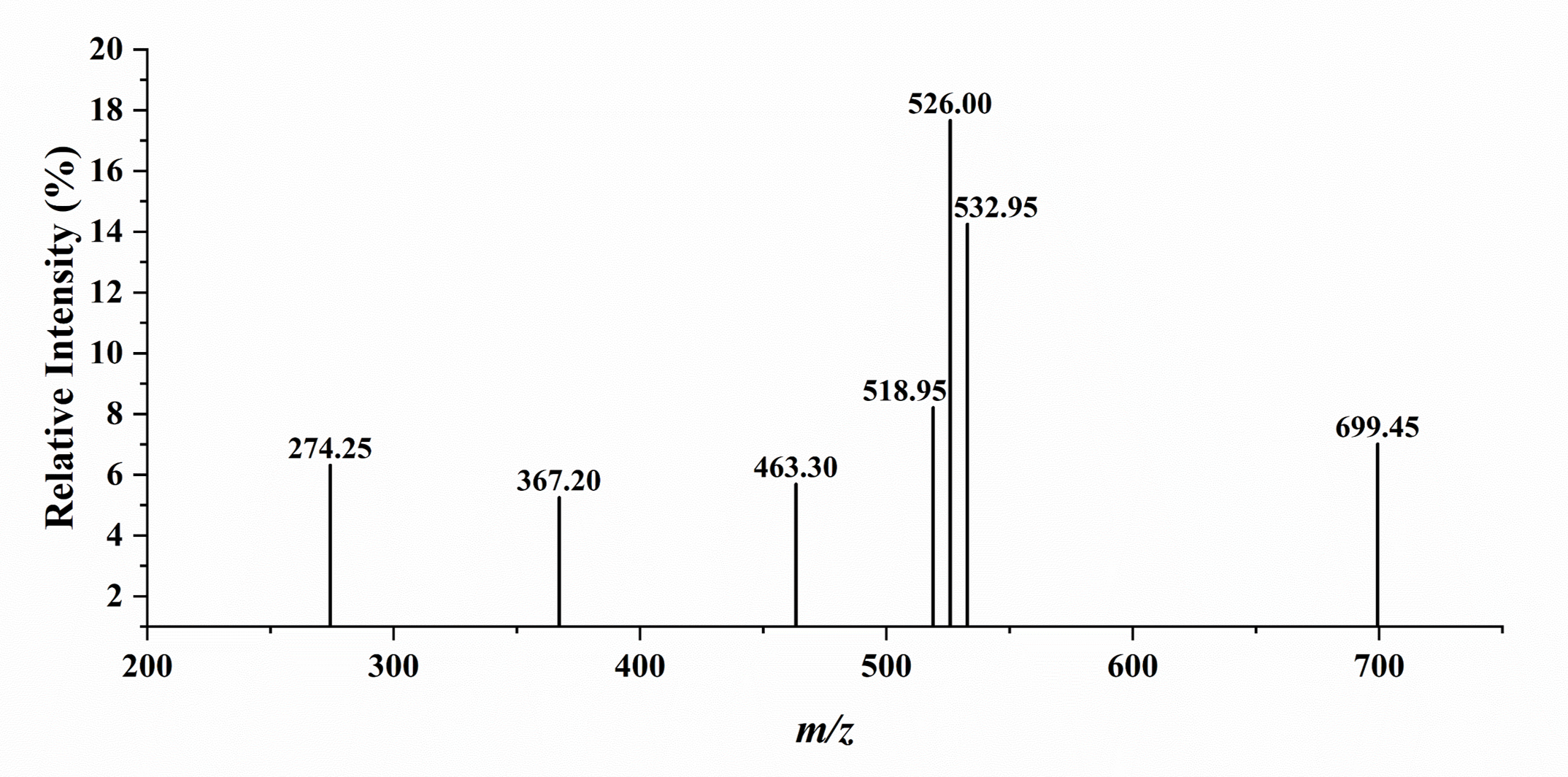

Supplement: Supplementary file 8 [file Image_7.TIF]
